# Change Points in the Population Trends of Aerial-Insectivorous Birds in North America: Synchronized in Time across Species and Regions

**DOI:** 10.1371/journal.pone.0130768

**Published:** 2015-07-06

**Authors:** Adam C. Smith, Marie-Anne R. Hudson, Constance M. Downes, Charles M. Francis

**Affiliations:** Canadian Wildlife Service, Environment Canada, Ottawa, Ontario, Canada; University of Regina, CANADA

## Abstract

North American populations of aerial insectivorous birds are in steep decline. Aerial insectivores (AI) are a group of bird species that feed almost exclusively on insects in flight, and include swallows, swifts, nightjars, and flycatchers. The causes of the declines are not well understood. Indeed, it is not clear when the declines began, or whether the declines are shared across all species in the group (e.g., caused by changes in flying insect populations) or specific to each species (e.g., caused by changes in species’ breeding habitat). A recent study suggested that population trends of aerial insectivores changed for the worse in the 1980s. If there was such a change point in trends of the group, understanding its timing and geographic pattern could help identify potential causes of the decline. We used a hierarchical Bayesian, penalized regression spline, change point model to estimate group-level change points in the trends of 22 species of AI, across 153 geographic strata of North America. We found evidence for group-level change points in 85% of the strata. Change points for flycatchers (FC) were distinct from those for swallows, swifts and nightjars (SSN) across North America, except in the Northeast, where all AI shared the same group-level change points. During the 1980s, there was a negative change point across most of North America, in the trends of SSN. For FC, the group-level change points were more geographically variable, and in many regions there were two: a positive change point followed by a negative change point. This group-level synchrony in AI population trends is likely evidence of a response to a common environmental factor(s) with similar effects on many species across broad spatial extents. The timing and geographic patterns of the change points that we identify here should provide a spring-board for research into the causes behind aerial insectivore declines.

## Introduction

Avian aerial insectivores (i.e., swallows, swifts, nightjars, and flycatchers; hereafter AI) are declining, and the causes of these declines are poorly understood. As a group, rates of long-term population decline for AI are similar or greater than declines in any other group of birds in North America [1]. For two regions of eastern Canada, population trends for many AI species became may have become more negative during the 1980s, i.e., the declines began or intensified during the 1980s [2]. If the declines intensified or were initiated in the 1980s, it would imply a group-level change point in population trends: that is, a change in population trends synchronized across many or all species in the group. If most, or all, AI species shared a common change point in their population trends, it suggests some common ecological or environmental cause behind the group’s decline, and that the timing and geographic patterns of that change point could help to identify potential causal factors in the group’s declines.

Studying population declines in the context of a group of species assumes that the population trends (which we define as average annual rates of change) and trajectories (defined more broadly as the pattern of temporal fluctuations in abundance) of species in the group reflect similar responses to one or more environmental or ecological factors. Even if all species in the group have responded in synchrony to some common factor, there may also have been species-specific responses to other environmental and ecological factors. More specifically, if there were change points in the population trends of AI, the changes may have been specific to each species, shared across all species in the group, shared across species within subgroups of AI (e.g., declines in FC may be caused by common factors that are distinct from the factors causing declines in SSN [2]), or most likely, some combination of species-specific and group-level. If the change points and their causes were only specific to each species, then the perceived group decline is coincidental, and further study in the context of the group is unlikely to be helpful. However, if change points and their causes are shared across species, then identifying those change points may help to identify the common factors which, if addressed, could benefit many species. Therefore, primary goal here is to assess the evidence in support of, and to describe the geographic pattern in, a group-level change point in aerial insectivore population trends.

The best way to test the hypothesis and estimate the timing of group-level trend change points is to directly model the group change points [3], [4], and not simply to compare the timing of species-specific change points. Species-level trajectories would not necessarily reflect a group-level change equally well, even if some past change in the environment did cause a group-level change in the trends of AI. The population trajectory for any given species will reflect both species-specific responses to environmental and ecological factors (e.g., disease, habitat change, and competition), as well as any change related to a group-level response. Indeed, in some cases, species-specific change points could confound group-level change points (e.g., a species-specific change could intensify, buffer, or reverse the effects of the group change). The change points that were previously identified for two regions of eastern Canada [2] may represent regions where species-specific changes coincided by chance, or where a group-level change point was particularly strong, or where few species-specific changes confounded the group pattern. Simple comparisons of species-specific change points cannot distinguish among these alternatives. Therefore, the best approach, and the one we take here, is to simultaneously model both group-level and species-specific change points for all species in the group, and to compare the support for that model to the support for a model that only includes the species-specific change points. Our approach is also an improvement over the earlier analyses in that our model accounts for the varying precision of annual indices across species, years, and regions, and so is much less sensitive to fluctuations in species trajectories that result from extreme and imprecisely estimated annual indices.

To use annual indices as data in a subsequent analysis of trend change points, or any non-linear aspect of a population trajectory, they should be derived from a model optimized to estimate the annual indices themselves (hereafter an “index model”), and not a trend model, which would assume a particular pattern of population change over time (e.g., a log-linear trend line). The indices of annual abundance from the North American Breeding Bird Survey (BBS), calculated annually by the United States Geological Survey (USGS) and Environment Canada’s Canadian Wildlife Service (CWS), are derived from a trend model and are not well-suited to an analysis focusing on change points. The models used by the USGS and the CWS are optimized to estimate long-term, average annual rates of change. The hierarchical structure of the year effects assumes that the deviations of the annual indices from a long-term trend line are random effects: zero-mean normally distributed parameter-distributions with a common variance across years and within geographic strata [5], [6]. The geographic strata in this and the USGS and CWS models are intersections of States, Provinces, and Territories—important management units—with Bird Conservation Regions (BCR)—biologically defined regions of North America with particular relevance to birds [5], [6]. Because they are random effects, the annual fluctuations are partially smoothed towards the trend line, if the data in a given year are relatively sparse. Because the level of smoothing is data-dependent, the complexity of the population’s trajectory varies among strata (i.e., strata with more routes support more complex trajectories) and among species (i.e., more common species support more complex trajectories). Similarly, the complexity of trajectories varies across the time series of a particular species and region, if the density of the data (e.g., the number of BBS routes run) fluctuates across years. In the context of population trend estimation, this smoothing characteristic of the model is desirable because it stabilizes trend estimates for species with relatively sparse data, while retaining the ability to model the annual variation well for species with relatively dense data [7]. However, if the ultimate objective is to model annual fluctuations or change points, this smoothing masks some of the variation of interest and even creates patterns in the population’s trajectory as data density changes over time (e.g., smooth trajectories in the early part of the BBS time series when fewer routes were run in any given year, and more complex trajectories in the later part of the time series [5]). Both trend models [5], [6], [8], [9] and index models [6], [10], [11] are commonly used in estimating the status and trends of bird populations in North America; however, the implications of these different model types and the consequences for subsequent analyses of the estimates are rarely discussed, although see [12], [13].

Our specific objectives in this study were to: 1) describe and fit an annual index model for BBS data that provides annual indices that are appropriate for use in a geographically structured analysis of trend change points, for as many species of AI as possible; 2) using these annual indices as data, fit another hierarchical Bayesian model to assess the evidence in support of group-level change points; 3) estimate the number and timing of group-level change points in AI population trends; and 4) make the annual indices and change point estimates available for further exploration and analysis by researchers seeking to explain AI declines.

## Results

Overall, we found evidence of synchronized changes in the population trends of multiple aerial insectivore species (i.e., group-level change points). Further study into the mechanism behind these changes in AI trends should help researchers understand the causes of aerial insectivore population declines.

### Estimating annual indices for aerial insectivores

By applying a hierarchical Bayesian, spatially explicit, intrinsic conditional autoregressive, annual index model (hereafter the spatial CAR model) to data from the North American Breeding Bird Survey, we estimated annual indices for the 22 most common and broadly distributed species of aerial insectivores (see Table 1 for full list and scientific names). Not all species of North American AI could be included because the spatial CAR model requires observations in most years within each stratum and many (>~10) strata overall to support the modelling of spatial year effects separately in each year. It also requires more data than does the trend model used annually by the CWS and USGS [5], [6].

**Table 1:** Species of Aerial Insectivores Included in the change point modeling, with Accompanying Scientific Names, Group Associations, and Summaries of the Species-Specific Change Points Identified.

					Species-specific Change Points				
Species	Scientific Name	Aerial Insectivore Group	Common Family Name	Number of Strata Included	Negative Change Points	Positive Change Points	Both Negative and Positive Change Points	At Least 1 Change Point	Percent of Included Strata With at Least 1 Change Point
Common Nighthawk	*Chordeiles minor*	SSN	Nightjars	71	32	1	1	32	45%
Eastern Whip-poor-will	*Antrostomus vociferus*	SSN	Nightjars	34	0	0	0	0	0%
Chimney Swift	*Chaetura pelagica*	SSN	Swifts	99	67	5	4	68	69%
Purple Martin	*Progne subis*	SSN	Swallows, Martins	93	20	15	6	29	31%
Tree Swallow	*Tachycineta bicolor*	SSN	Swallows, Martins	91	27	38	11	54	59%
Violet-green Swallow	*Tachycineta thalassina*	SSN	Swallows, Martins	30	0	5	0	5	17%
Northern Rough-winged Swallow	*Stelgidopteryx serripennis*	SSN	Swallows, Martins	106	1	20	0	21	20%
Barn Swallow	*Hirundo rustica*	SSN	Swallows, Martins	150	81	57	36	102	68%
Olive-sided Flycatcher	*Contopus cooperi*	FC	Tyrant Flycatchers	36	23	9	9	23	64%
Western Wood-Pewee	*Contopus sordidulus*	FC	Tyrant Flycatchers	38	1	3	0	4	11%
Eastern Wood-Pewee	*Contopus virens*	FC	Tyrant Flycatchers	97	8	22	1	29	30%
Yellow-bellied Flycatcher	*Empidonax flaviventris*	FC	Tyrant Flycatchers	10	4	0	0	4	40%
Acadian Flycatcher	*Empidonax virescens*	FC	Tyrant Flycatchers	52	2	9	0	11	21%
Alder Flycatcher	*Empidonax alnorum*	FC	Tyrant Flycatchers	40	5	28	4	29	73%
Willow Flycatcher	*Empidonax traillii*	FC	Tyrant Flycatchers	60	9	2	0	11	18%
Least Flycatcher	*Empidonax minimus*	FC	Tyrant Flycatchers	52	17	5	1	21	40%
Pacific-slope Flycatcher	*Empidonax difficilis*	FC	Tyrant Flycatchers	11	3	1	1	3	27%
Eastern Phoebe	*Sayornis phoebe*	FC	Tyrant Flycatchers	95	57	85	57	85	89%
Say's Phoebe	*Sayornis saya*	FC	Tyrant Flycatchers	33	10	5	4	11	33%
Great Crested Flycatcher	*Myiarchus crinitus*	FC	Tyrant Flycatchers	101	3	20	0	23	23%
Western Kingbird	*Tyrannus verticalis*	FC	Tyrant Flycatchers	53	12	24	8	28	53%
Eastern Kingbird	*Tyrannus tyrannus*	FC	Tyrant Flycatchers	127	56	13	8	61	48%

**Fig 1:**
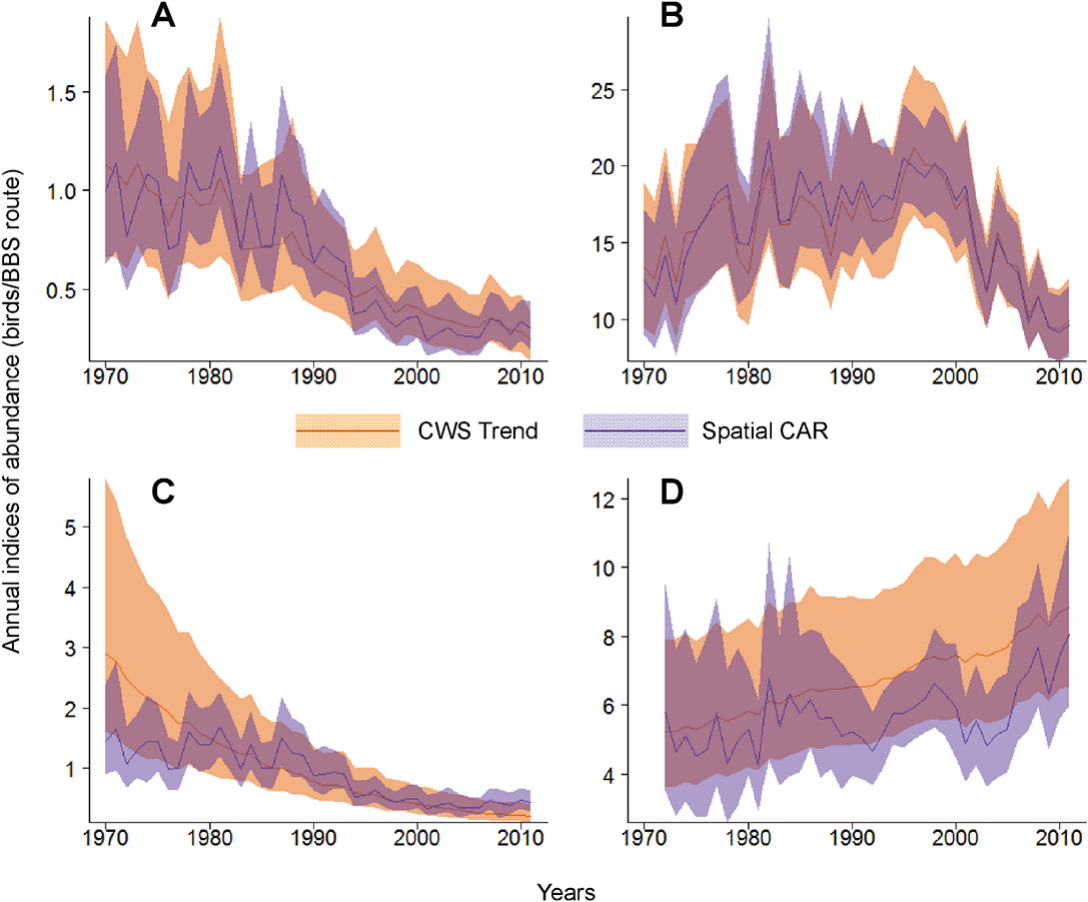
Comparison of Annual Indices of Abundance from the BBS Generated by the Annual Index Model (Spatial CAR) and by the Trend Model Currently Used by Environment Canada’s Canadian Wildlife Service (CWS Trend). Examples include plots for Olive-sided Flycatcher in Ontario/Bird Conservation Region (BCR)12 (A), Tree Swallow in Ontario/BCR 13 (B), Olive-sided Flycatcher in Quebec/BCR 12 (C), and Tree Swallow in Alberta/BCR 6 (D). Species and regions represent examples where the two models give very similar estimates (e.g. A and B), or very different estimates (e.g., C and D). Region names relate to analytical strata, which are defined by the intersections of Canadian provinces with Bird Conservation Regions (BCRs, indexed here by their numbers). BCR names are: BCR 12, Boreal Hardwood Transition; BCR 13, Lower Great Lakes / St. Lawrence Plain; and BCR 6, Boreal Taiga Plains.

As expected, trajectories from the trend models used by the USGS and CWS were more linear (i.e., showed fewer cycles and fluctuations) than trajectories generated by the spatial CAR model. In some strata and for some species (e.g., Olive-sided Flycatcher and Tree Swallow), there were only minor differences between the two models, particularly when there were many routes and years with observations in a given stratum (Fig 1A and 1B). By contrast for some strata and species, annual indices from the trend model were smoothed toward the estimated long-term trend in the population, and did not show the cycles or changes that were evident in the estimates from the spatial CAR model (Fig 1C and 1D).

### Support for grouping aerial insectivores

**Fig 2:**
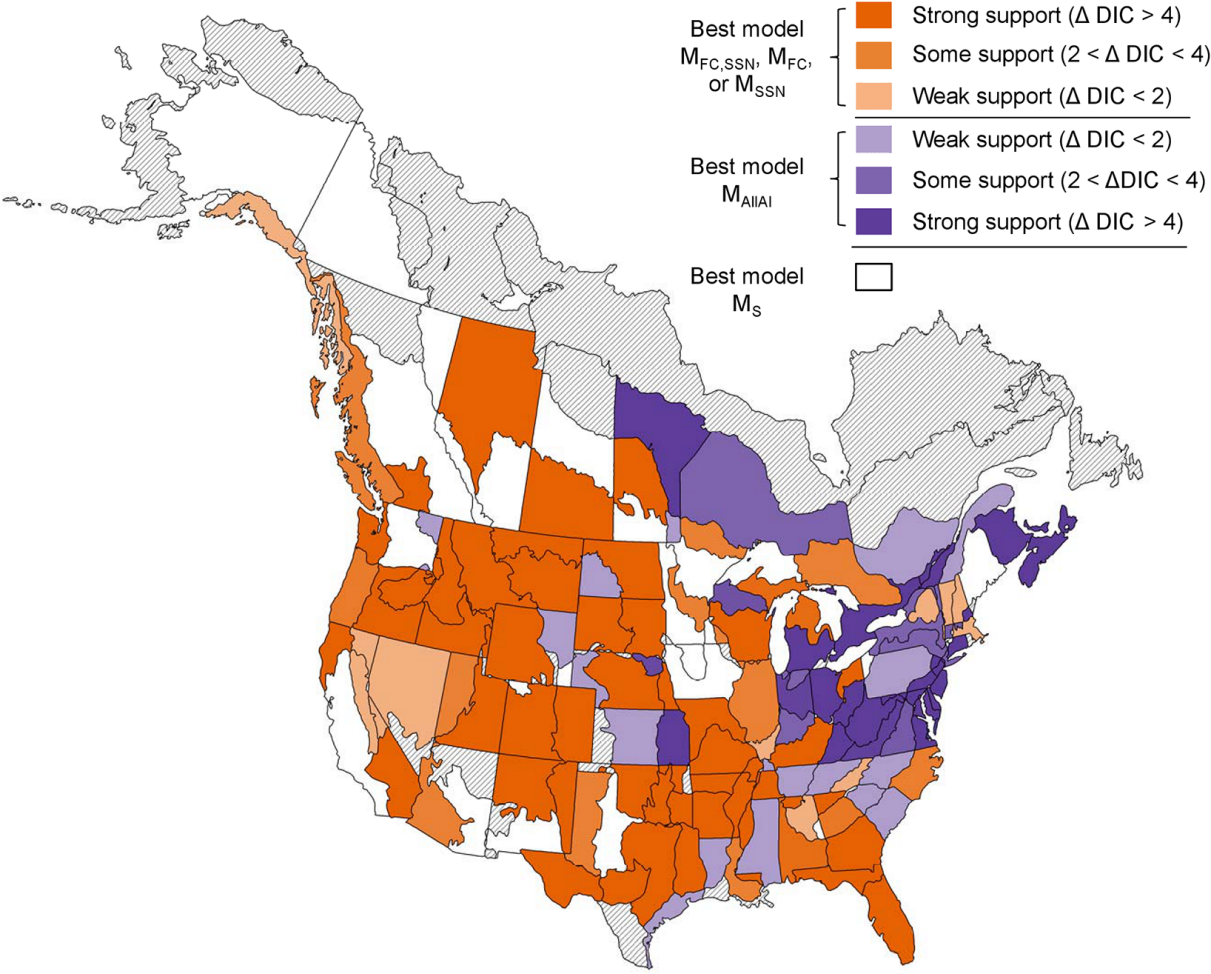
Geographic Distribution of the Relative Support for 5 models Testing Shared Population Trend Changes among North American Aerial Insectivores. These models include a common group change point for all aerial insectivore species (strata in shades of purple, best model M_allAI_), separate group change points for flycatchers (FC) and swallows, swifts, and nightjars (SSN; strata in shades of orange, best model one of M_FC,SSN_, M_FC_, or M_SSN_), or no group change point (strata in white, best model M_S_). Intensity of the purple or orange reflects the relative support for the best model over the next best model represented by another colour (e.g., the best model in the dark orange coloured strata has DIC at least 4 units less than the DIC for either M_AllAI_ or M_S_).

Using the annual indices from the spatial CAR model as data for the Bayesian piecewise linear regression spline model, we found broad support for group-level change points in the population trends of North American aerial insectivores. In 85% of the 153 geographic strata for which we had sufficient data to run a model (annual indices for 2 or more species of both SSN and FC), the best supported model (lowest DIC) included at least one group change point (M_AllAi_, M_FC,SSN_, M_FC_, or M_SSN_ (Table 2); coloured various shades of either purple or orange; Fig 2, S1 Table). The few remaining strata where the model with no group change point (M_s_) was best supported (coloured white; Fig 2) were predominantly central and in the west. In these cases, the relative support for the M_s_ model over the best supported group change point model was relatively weak (mean ΔDIC = 2.4, range [0.03, 5.2]; S1 Table).

**Table 2:** Model Names, Equations and Associated Interpretations for each of the 5 Change Point Models.

Model	Equation	Model interpretation
M_s_	${\text{H}}_{i,t,s}=Normal\left(f_{i,s}\left(t\right),\sigma_{p}^{2}\right)$	Changes in AI populations are species-specific; group-level patterns are likely coincidental.
M_AllAI_	${\text{H}}_{i,t,s}=Normal\;(f_{i,s}\left(t\right)+f_{i,AllAI}\left(t\right),\sigma_{p}^{2})$	Changes in AI populations are similar across all species in the group.
M_FC,SSN_	${\text{H}}_{i,t,s}=Normal\;\left(f_{i,s}\left(t\right)+f_{i,FC}\left(t\right)+f_{i,SSN}\left(t\right),\sigma_{p}^{2}\right)$	Changes in AI populations are similar among flycatchers and among swallows, swifts, and nightjars, but are distinct between the two groups.
M_FC_	${\text{H}}_{i,t,s}=Normal\;\left(f_{i,s}\left(t\right)+f_{i,FC}\left(t\right),\sigma_{p}^{2}\right)$	Changes in swallow, swift, and nightjar populations are similar among species; changes in flycatcher populations are species-specific.
M_SSN_	$H_{i,t,s}=Normal\;(f_{i,s}\left(t\right)+f_{i,SSN}(t),\sigma_{p}^{2})$	Changes in flycatcher populations are similar among species; changes in swallow, swift, and nightjar populations are species-specific.

The best supported group change point models showed some broad geographic patterns (Fig 2). Across most of the continent, the data most often supported models that suggest FC species and SSN species showed separate change points (i.e., M_FC,SSN_, M_FC_, or M_SSN_; Fig 2, Table 2). However, in most strata of northeastern North America, the data supported a group change point in trend that was common to all aerial insectivores (M_AllAI_).

For all further results, we estimated the timing of group-level change points using model M_FC,SSN_ because it was the best supported model in most strata, and doing so allowed us to compare the geographic variation in change points while keeping species composition of the groups relatively constant.

### Change points for swallows, swifts, and nightjars

**Fig 3:**
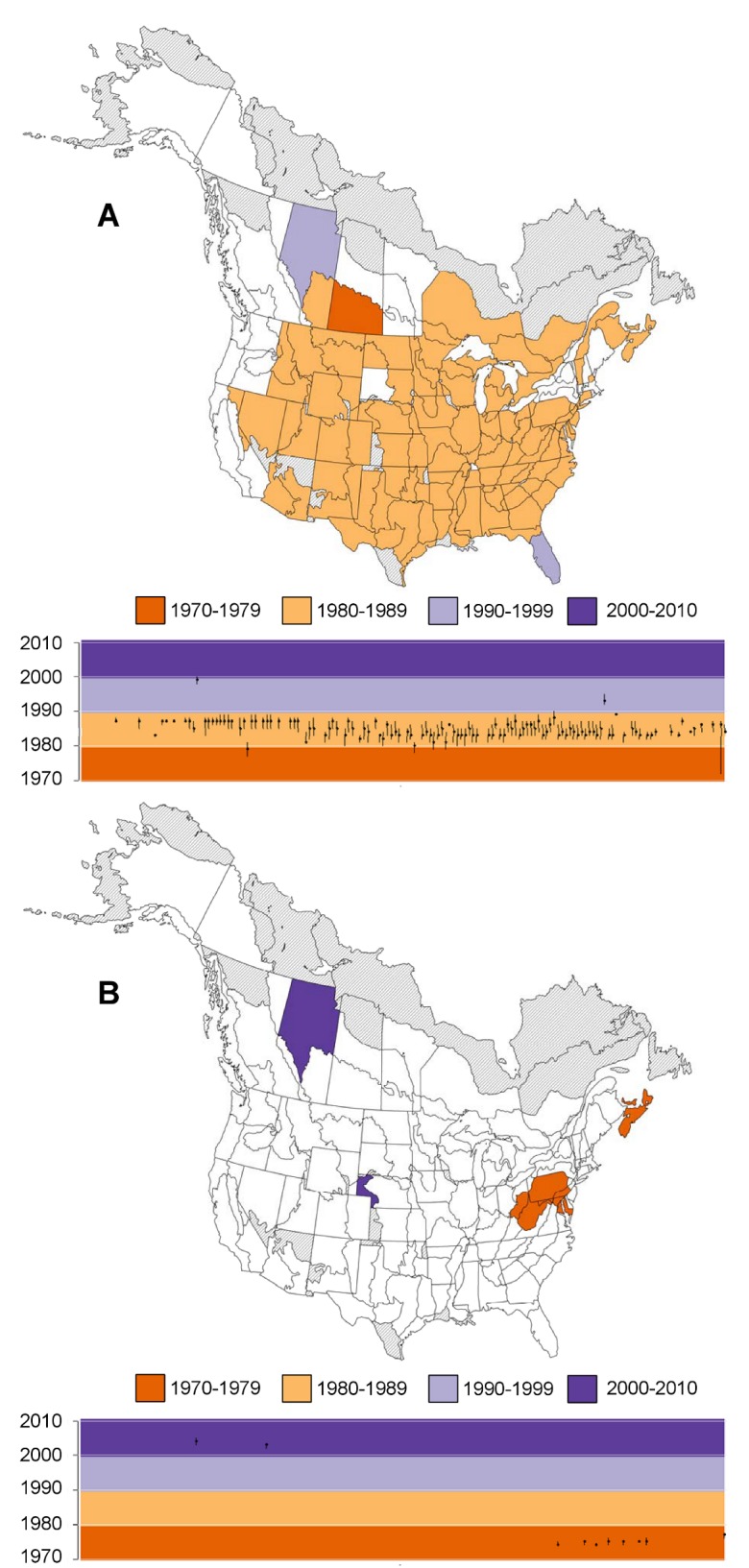
Timing of Well-Supported, Negative (A) and Positive (B) Change Points in Population Trends that are Shared across North American Swallows, Swifts, and Nightjars. Timings were estimated separately within each of the geographic strata; strata are coloured based on the year that had the highest posterior probability of including a change point. The plots below the maps show the years with highest probability (points) as well as adjacent years that also had relatively high posterior probability of including the change point (error bars, years with posterior:prior odds ratio > 3). In the plots, the strata are sorted from west to east based on the longitude of their centroid, and coloured regions reflect the decadal colours used in the maps. Strata coloured grey were not modeled due to insufficient data. Strata in white had no well-supported change points (either positive or negative).

**Fig 4:**
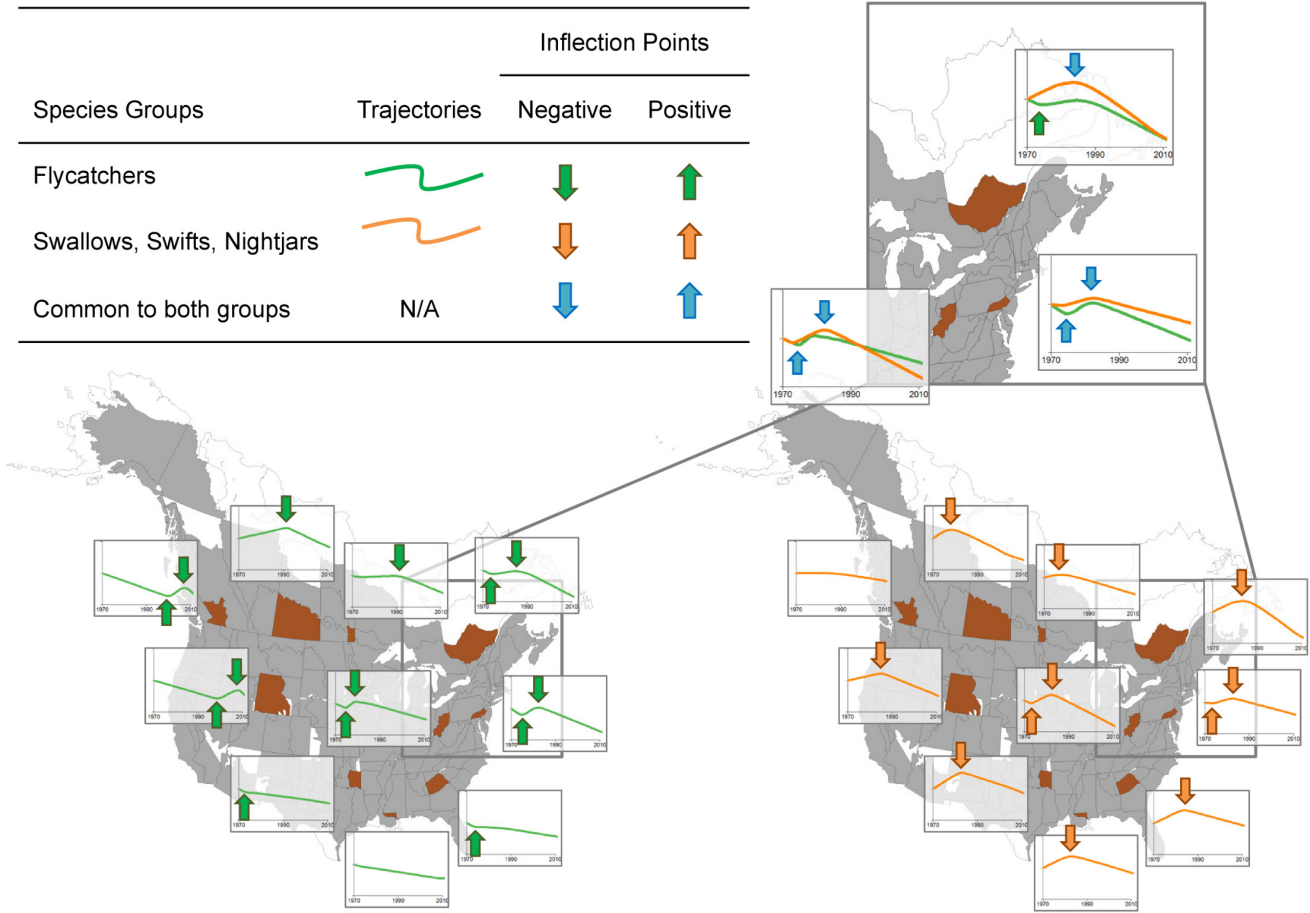
Examples of Group Trajectories (lines) and Well Supported Change Points in Trends (arrows) for North American Aerial Insectivores. Group trajectories are shown for a selection of strata (brown shaded stratum closest to each trajectory graph) that are broadly representative of group trajectories in the surrounding region. Group trajectories in these selected strata demonstrate that the two groups of aerial insectivores, FC (in green on left) and SSN (in orange on right), have followed largely separate trajectories over much of North America. The exception is in the northeast, where the two groups of aerial insectivores have followed relatively similar trajectories (inset).

Across most of North America, swallows, swifts, and nightjar population trends showed a common, well-supported negative change point during the 1980’s (Fig 3A). There were a few strata where the data supported a positive change point in SSN trends. The majority of these (few) positive change points were in eastern North America; in almost all cases, they occurred a few years prior to the negative change point (Fig 3B). Overall, SSN trajectories appeared very similar across the continent due to the single negative change point that was consistent across most strata. However, the magnitude and direction of the group trends, (i.e., the slopes before and after the common change point) showed some geographic variation. In the west and northeast, the trends were initially moderately positive or stable, followed by a relatively steep decline through to the present (orange trajectories in Fig 4). In the south, many trajectories began with a period of steep increase, followed by a decreasing or stable trajectory through to the present (Fig 4). This geographic pattern in the trends before and after the change points resulted in some geographic variation in the overall trend among strata (greater overall decline in the northeast and less overall decline in the south), even though the timing and direction of the change point was consistent across the strata.

### Change points for flycatchers

**Fig 5:**
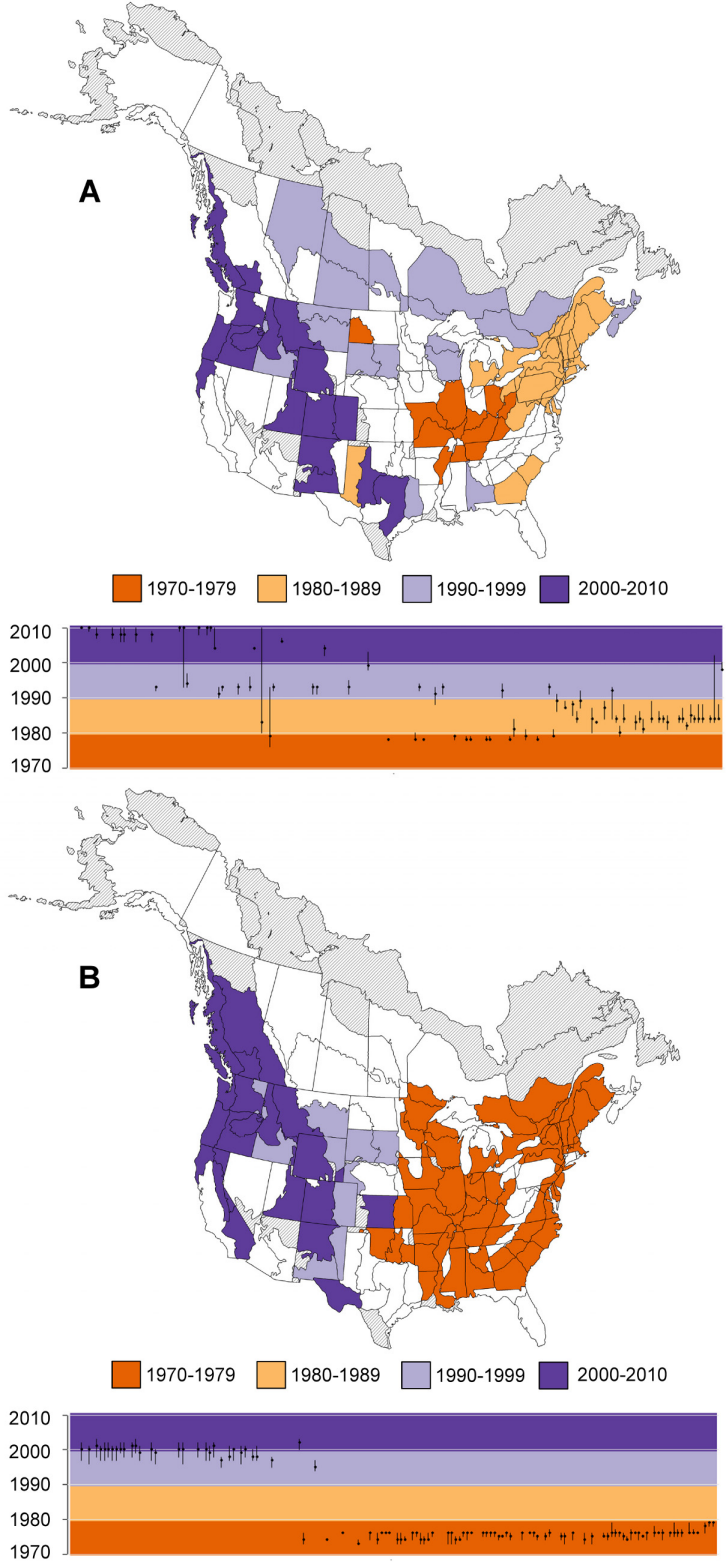
Timing of Well-Supported Group-Level, Negative (A) and positive (B) Change Points in Population Trends that are Shared across North American Flycatcher Populations. Timings were estimated separately within each of the geographic strata; strata are coloured based on the year that had the highest posterior probability of including a change point. The plots below the maps show the years with highest probability (points) as well as adjacent years that also had relatively high posterior probability of including the change point (error bars, years with posterior:prior odds ratio > 3). In the plots, the strata are sorted from west to east based on the longitude of their centroid, and coloured regions reflect the decadal colours used in the maps. Strata coloured grey were not modeled due to insufficient data. Strata in white had no well-supported change points (neither positive nor negative).

Flycatcher population trajectories showed common, well-supported negative change points in many strata across North America, but the timing of these downturns varied geographically (Fig 5A). In general, the negative change points occurred in the late 1970s in parts of the lower American Midwest, Kentucky, and Tennessee; in the mid-1980s in the northeastern US, parts of Texas, Georgia and South Carolina, and eastern Canada (with the exception of Nova Scotia); in late 1980s and early 1990s in much of the rest of Canada, north-central US, and parts of Texas and Alabama; and in the 2000s in the western US, western British Columbia, and Alaska. Flycatcher population trajectories in many strata also showed consistent positive change points that were strongly geographically separated: occurring either in the 1970s in eastern North America, or approximately in 2000 in western North America (Fig 5B). In the west, FC trajectories were characterized by a steep, long-term decline from the start of the time series (1970), followed by a positive change point in the late 1990s or early 2000s, a period of population increase, and finally a negative change point and decrease starting approximately in 2009 (Fig 4). In the southeast, FC trajectories were broadly similar to trajectories in the northeast for the early part of the time series, but in most strata, the only clear change in trend was an early, positive change point that moderated the previously steep rate of decline (Fig 4).

### Change points common to all aerial insectivores

In northeastern North America, the two groups of aerial insectivores have followed relatively similar trajectories (purple strata in Fig 2 and examples in Fig 4, inset). The group-level trajectory generally included an initial period of decline, followed by a positive change point and a short period of increase, then a negative change point in the mid-to-late 1980s that started the long-term decline that continues today (examples in Fig 4). Although there is some variation in the level of support for particular change points when group trajectories were estimated separately for FC and SSN (i.e., contrasts between Figs 3A and 5A, and between Figs 3B and 5B), in most northeastern strata, the data showed relatively strong support (ΔDIC > 4) for a common change point across all aerial insectivores (Fig 2 and S1 Table). In addition, when estimated separately, the negative change points for each group occurred at very similar times (mid-to-late 1980s) in the northeastern strata (similar orange and green trajectories in of the inset of Fig 4). Furthermore, in the few strata showing a well-supported positive change point for SSN, the timing of the positive change point was coincident with the positive change point estimated separately for FC in the region (purple strata in Figs 3B and 5B, and the positive change points identified by blue, upward pointing arrows in Fig 4).

We considered a posterior:prior odds ratio > 3, in any given year, as strong evidence in support of a trend change point. In each stratum and for each group, the year with the highest posterior probability and therefore the highest odds ratios, of a particular change point is identified by the points in the graphs of Fig 3 and Fig 5. In many regions, the odds ratio exceeded 3 in more than 1 year, reflecting some uncertainty in the precise timing of the group-level change point (error bars in the graphs of Figs 3 and 5 and S1 Table). This uncertainty in the timing combined with variation in the posterior probabilities, of a trend change point in any given year (S1 Table), complicates a concise summary of the uncertainty around the group-level change points that we’ve identified. We summed the posterior probabilities across all sequential years in which a particular group-level change point was strongly supported, as a measure of the overall support in the data for that change point. By this measure, negative change points during the 1980s, for both SSN and FC, as well as positive change points for FC during the 1990s and 2000s, generally had the most overall support in the data (median > 0.73, S1 Table and S1 Fig) when summarized across all strata with a change point in each decade.

### Magnitude of the change points

**Fig 6:**
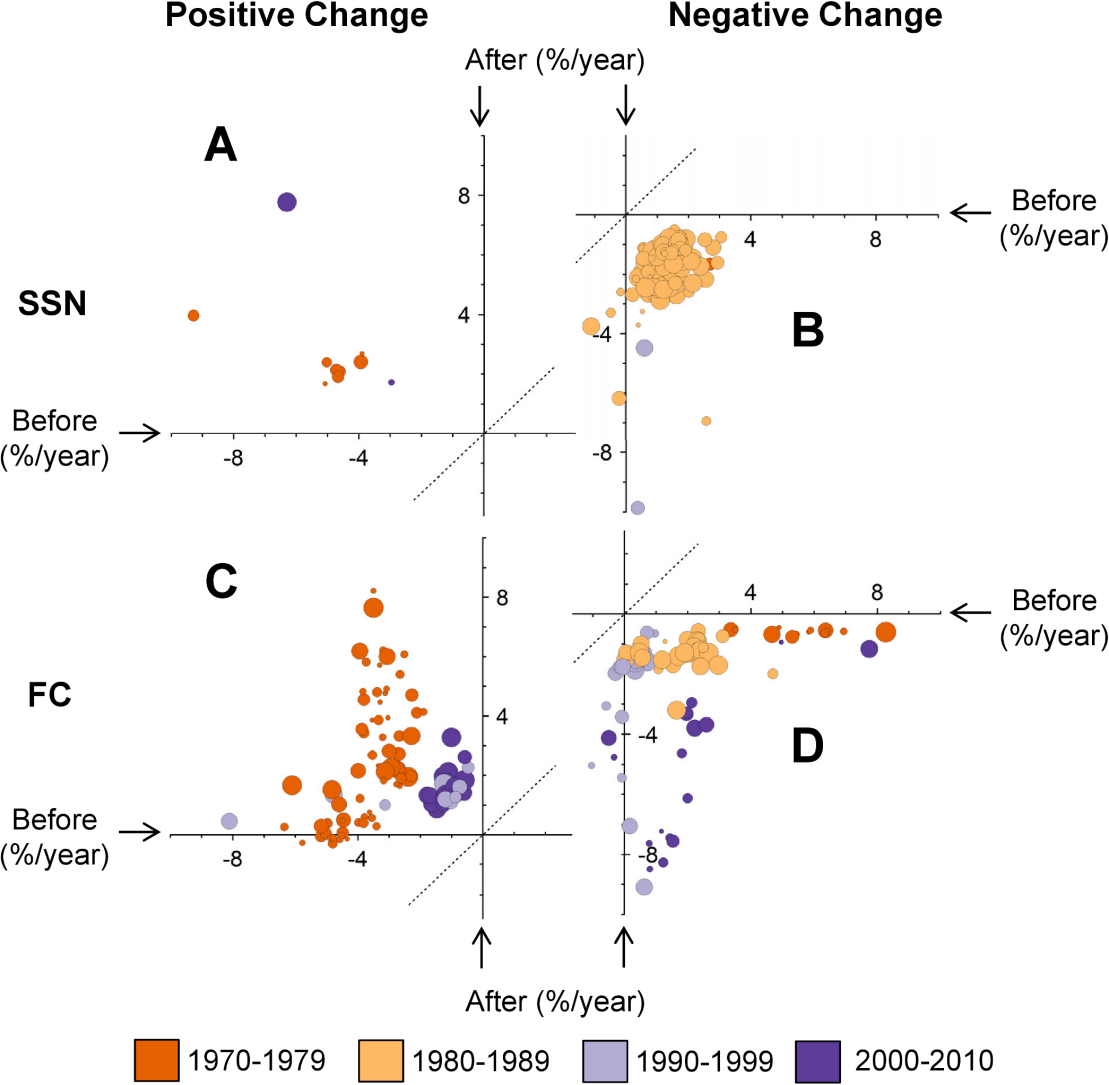
Group-level Trends for Flycatchers (FC) and for Swallows, Swifts, and Nightjars (SSN), Before and After Well-Supported, Group-Level Change Points. Group-level trends are defined as the average annual rates of change from the group-level trajectories of the piecewise linear regression spline model. The graph is divided into positive (A and C) and negative (B and D) change points for species of swallows, swifts, and nightjars (A and B), and flycatchers (C and D). Only one quadrant from each of the four plots is shown: the quadrant that included points representing all well-supported change points for a particular group and direction. The colours of the points indicate the decade in which the change point occurred and match previous figures (Figs 3 and 5). The size of the points reflects the posterior probability of the change point occurring at that time (i.e., larger points reflect change points with higher probability).

In the regions with well-supported positive change points for SSN, the group trends were all strongly negative before the change point and positive afterwards (Fig 6A). At the change point, the rate of population change increased by approximately 6%/year (from approximately 4%/year decline to 2%/year increase). In regions with well-supported negative change points for SSN, the group trends were positive, relatively stable, or slightly negative (between 1%/year decline and 3%/year increase) before the change point and were all negative following the change points (Fig 6B). At these negative change points, most of the trends decreased by approximately 2–4%/year, although a few decreased by much more.

In regions with well-supported positive change points for FC, the change points that occurred in the east during the late 1970’s (Fig 6C, where trends increased from declines of 2–6%/year to increasing trends of up to 8%/year) were greater in magnitude than the positive change points that occurred in the west during the 2000’s (dark purple points in Fig 6C, where trends increased from declines of approximately 1%/year to increases of 1.5%/year). In the regions with well-supported negative change points for FC, the change points in the 1970s (dark orange circles in Fig 6D) were changes from steeply increasing populations to relatively stable populations (from increases of 4–8%/year to declines of 0.5%/year), those in the 1980s (light orange circles) were changes from increasing or stable populations to declining populations (from increases of 0–3%/year to declines of 1–2%/year), those in the 1990s (light purple circles) were relatively stable populations that were all declining afterwards (from trends near 0 to declines of 1–9%/year); and those in the 2000s (dark purple circles) were mostly increasing populations that were all declining afterwards (from increases of 1–2%/year to declines of 1–9%/year).

### Species-specific change points

**Fig 7:**
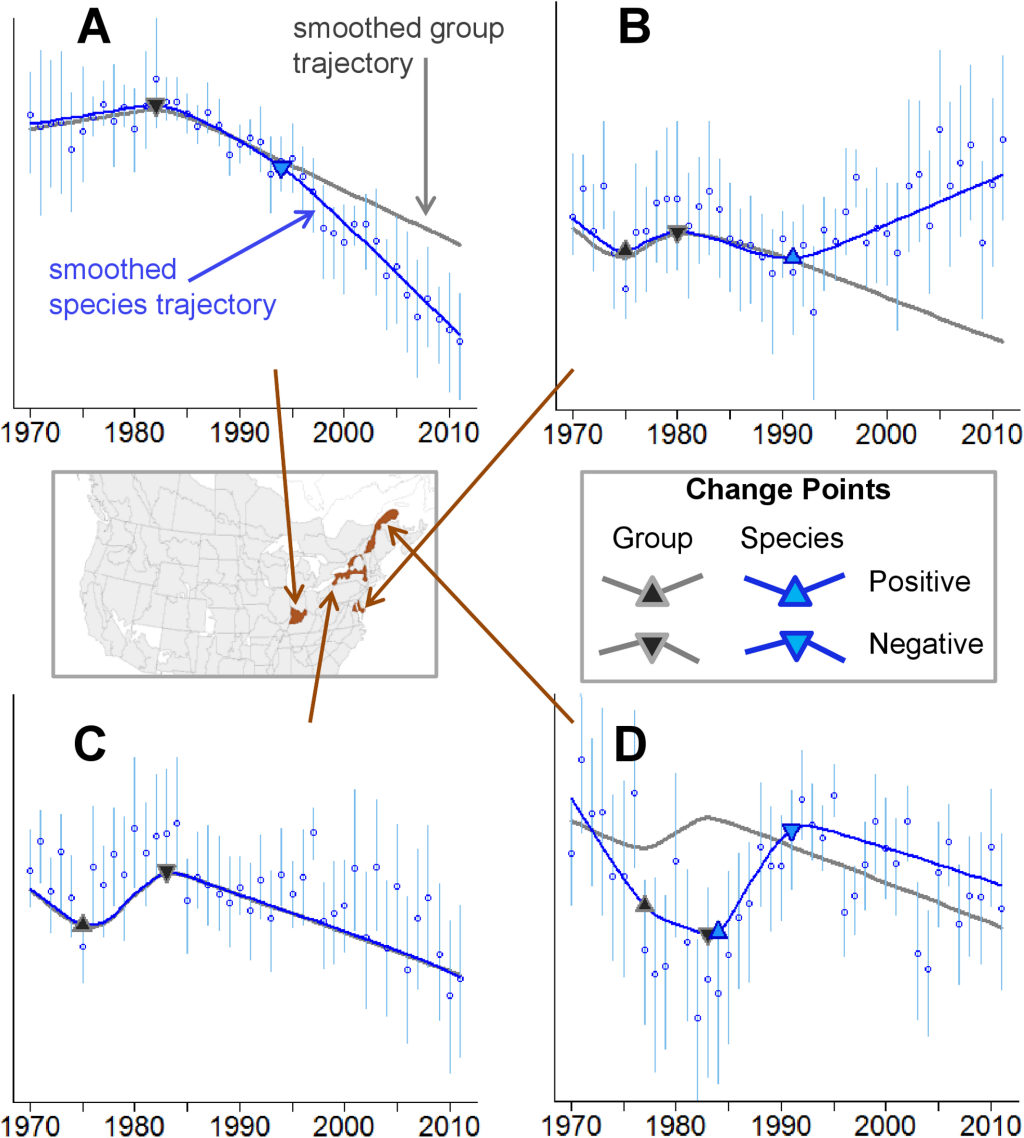
Examples of Simultaneously Estimated, Species-Specific (Blue Triangles) and Group-Level (Black and Grey Triangles) Change Points, for a Selection of Species and Regions. Triangles pointing up represent positive change points, while triangles pointing down represent negative change points. Many species’ trajectories follow the group trajectory relatively well for part of the time series, but then diverge following a species-specific change point: Chimney Swift in the Indiana portion of Bird Conservation Region (BCR) 24 (A), and Great Crested Flycatcher in the Maryland portion of BCR 30 (B). Many other species trajectories include only the group-level change points: Great Crested Flycatcher in the New York portion of BCR 13 (C). In a few cases the species-specific change points contradict the group-level change points: Eastern Phoebe in the Quebec portion of BCR 14 (D). Region names relate to analytical strata, which are defined by the intersections of states and provinces with BCRs. BCR names are: BCR 13, Lower Great Lakes / St. Lawrence Plain; BCR 14, Atlantic Northern Forest; BCR 24, Central Hardwoods; and BCR 30, New England/Mid-Atlantic Coast.

In addition to the group-level change points that we have focused on here, we also identified species-specific change points. For all species included in our analyses, except Eastern Whip-poor-will, species-specific change points were identified in at least one stratum (Table 1). Species varied in the number and direction of these species-specific change points. For example, Western Wood-Pewee, Acadian Flycatcher, and the Northern Rough-winged Swallow had species-specific change points in relatively few of the strata where they were included (Table 1). By contrast, Chimney Swift, Eastern Phoebe, Tree Swallow, Olive-sided Flycatcher, and Barns Swallow had species-specific change points in many (up to 89% of the strata where they were included, Table 1). In almost all cases, the species-specific change points represented change points that followed or pre-dated the group-level change points (Fig 7A and 7C). However, for Eastern Phoebe, there was a positive species-specific change point that directly contradicted the group-level negative change point that occurred in Eastern North America (Fig 7D, Table 1). We have provided a table detailing the timing of positive and negative species-specific change points, for all species included here (22 species across 153 strata), in S2 Table to enable further study by researchers and managers interested in single species of AI.

## Discussion

Our results show that there have been numerous change points in the population trends of aerial insectivorous birds and that many change points were shared across most or all species in the group. In particular, we found strong support for a negative change point in AI population trends in the 1980s, which was previously identified for some regions of eastern Canada [2]. Also, our findings greatly extend this earlier work, showing that the change points are generally separate for two groups of AI (i.e., FC versus SSN) and that for SSN species, the negative change point in the 1980s extends across almost all of North America. It is even evident in regions of the continent (e.g., the southeast), where to date, there has been little concern over declines in AI populations [2].

The fact that there are strongly supported group-level change points for aerial insectivores suggests that these species can be meaningfully grouped in their responses to changes in the environment, and that FC should generally be considered separately from SSN (Fig 2). The one exception was in northeastern North America, where the change points for both groups coincided. This could arise if both groups in this region are being affected by the same environmental factors. Alternatively, this may indicate that the factors that influence FC and SSN happened to change at the same time in the northeast.

Although the steep negative population trends for AIs places a natural emphasis on negative change points, our work shows that positive trend change points are present in many aerial insectivore population trajectories. The existence of these positive change points suggests that research into the causes of decline should not only examine the negative change point (i.e., the times at which aerial insectivore declines began, or intensified), but also the positive change points. For example, what caused eastern flycatcher populations to increase during the late 1970s and early 1980s, and the western populations to increase in the late 1990s and early 2000s? Given that many of the group trajectories show declines both before and after these brief periods of increase (Fig 5), the negative change points may not necessarily signify a new change in the environment that initiated the group’s decline. Rather, since many FC and some SSN were in decline when the BBS began, the most recent negative change points could be interpreted as the end of a brief, relatively productive period.

Our results should be robust to numerous sources of uncertainty surrounding change point estimates for a group of species. First, by contrasting models with and without group-level change points, a single group-level change point would only provide a more parsimonious explanation than a collection of species-level change points if all or most of the species in the group contained a similar change point. Second, all our models accounted for the varying precision of the annual indices among years, regions and species, so we are confident that the change points we’ve identified reflect real changes in species’ trends and not coincidental sampling noise. Third, we chose to focus our analysis on the most significant changes in medium- to long-term trends by limiting each combination of species and group to a maximum of 4 change points. Finally, by simultaneously modeling species-level and group-level change points, we were able model the similarities among species trajectories, while accounting for species-specific departures from the group-level patterns.

We have focused here on the group-level change points that are shared among many species of AI, but in many cases there were also species-specific change points. For a given species, the group or species-specific factors may be more or less important in explaining the population’s current status and trend. Some species, such as Eastern Whip-poor-will, Northern Rough-winged Swallow, and the Western Wood-pewee, have relatively few, or no, change points beyond the group change points (Table 1). These species fit the group pattern of change very well and also tend to have less precise annual index estimates (i.e., less precise data), which generally provide weaker support for change points than more precisely estimated annual indices. Other species fit the group pattern very well, but show additional strong change points earlier or later in the time series. For example, the annual indices and species-specific spline for Great Crested Flycatcher in most of BCR 13 fit the group-level change points and spline very well (Fig 7C). By contrast, the group trajectory and change points in BCR 30 are almost identical to those in BCR 13, but the species-specific spline includes a strong positive change point in the early 1990s (Fig 7B), which reverses an earlier population decline. Similarly, in many strata, the Chimney Swift shows a species-specific negative change point in the 1990s (Fig 7A). Some species, particularly the Eastern Phoebe, have change points that directly counteract the group change point (i.e., the change points occur at the same time but in the opposite direction, Fig 7D). These species-specific departures from the group patterns may represent noteworthy opportunities for more focused studies into the mechanisms underlying group and species-specific changes. Identifying—and eventually reversing or ameliorating—the factors that are causing declines in AI will likely require management directed at both the group-level and species-specific factors.

### Annual index models versus trend models for population status and trend

No single model is ideal for estimating trends and annual indices, so the parameters of greatest interest should determine the choice of the most appropriate model. Given consistent and balanced sampling, annual index models and trend models should generate similar estimates for both trends and annual indices. However, under realistic expectations for a continental-scale, volunteer-based survey, sampling will always be unbalanced to some degree. If modeled estimates are to be used as data in other analyses, users of existing trend or annual index estimates should be aware of the assumptions and structure of the model that produced the estimates. For example, the annual indices we have supplied here (or those derived from the similar model described by [10]) would not be ideal for contrasting trajectories between neighbouring strata for a given species, because of the imposed spatial autocorrelation in the spatial CAR model. Similarly, trend estimates derived from the model used here may differ from estimates derived from the trend model currently used to monitor population status and trends by the USGS [5] and CWS [6], because of the temporal smoothing behaviour of the trend model. Although differences among modeled estimates of population status and trends raise practical, philosophical, and political complications for species’ conservation status assessments [12], [13], the variation among estimates should serve as a reminder to users that model-based estimates are always conditional on the structure and assumptions of the model used to produce them. The BBS trends and annual indices, produced each year by the CWS and USGS, are invaluable for assessing the population status and trends of more than 400 North American bird species. However, they are not necessarily appropriate data in supplementary analyses, if the features of interest in the supplementary analysis (e.g., the annual fluctuations) are strongly influenced by an assumption of the original model.

### Future research to identify causes

The change points we have identified should be helpful in focusing future analyses of spatial and temporal covariates that might help to explain some of the widespread and drastic declines in aerial insectivore populations. For example, the positive change point for flycatchers in eastern North America suggests that the late 1970s and early 1980s saw an improvement in some factor(s) that had a relatively consistent effect on flycatcher species. However, in most regions, the effect was short-lived, and a negative change point followed anywhere from 3–10 years later. Similarly, the negative change points in swallow, swift, and nightjar trends across almost all of North America suggest that something exerting a consistent effect on these populations changed for the worse in the 1980s. Changes in land use [14], pesticides [15], and weather and climate [16] are known to affect at least one species of aerial insectivore, and there are likely to be many factors at play. So, not only will researchers have to tease apart the complicated interactions among these and other as-yet unknown factors, but the spatial and temporal variation in those interactions as well. Our results provide real, broad-scale patterns that researchers can focus on to begin tackling this daunting task.

The existence of such broadly consistent patterns in the timing and direction of change points suggests that there are powerful and broad-scale factors at work. These change points reflect broad patterns of group population change that span, and interact with, geographic variation in local environmental factors, species composition, migratory behaviour, and wintering distributions, to name but a few. For example, the strong east-west differences in the timing of flycatcher positive change points (Fig 5B) may be partly a function of differences in the species that primarily/exclusively occur in the west or east (S2 Fig), effects of large-scale environmental factors or gradients (e.g., Southern Oscillation and El Niño, North Atlantic Oscillation, and Pacific/North American teleconnection pattern, [17]), and/or differences in migratory pathways or wintering grounds used by populations with primarily eastern and western distributions (S2 Fig) [18], [19], [20].

The annual indices (S2 Table) and the models (S1 and S3 Texts) included here should be useful tools to discern among the numerous possible factors affecting these species. The change point model (S3 Text) can be extended to identify covariates that might explain these group and species-specific change points (e.g., [4]). With a relatively minor modification, the change point model could test the ability of annual covariates to explain the change points in the group trajectory and/or the species-specific change points by matching similar change points in the temporal trends of AI and the covariates. It would also be worth exploring further modifications to the change point model, or alternative models that could analyse the effect of a relatively abrupt change in a covariate (e.g., a step-change) on a gradual change in population status (e.g., a rate-parameter such as the trend between change points). Relevant covariates might include annual environmental factors measured on the breeding range, on the wintering range, within each of the analytical strata, or even among different parts of each species’ wintering range, assuming the wintering regions could be clearly linked to the species’ population in each analytical stratum. Similarly, demographic data (e.g., Monitoring Avian Productivity and Survivorship data [21], or nest monitoring data [www.nestwatch.org]) could be used as a covariate that could help identify the ecological processes (e.g., changes in survival, nest success, etc.) that may underlie the changes in population trends.

## Materials and Methods

### Modeling aerial insectivore trajectories across North America

For our objectives, we derived annual indices of abundance from a model optimized to estimate the annual indices themselves (an index model), and not published indices derived from a trend model [3], [4]. However, there are costs to abandoning the simplifying assumption of smooth population change underlying a trend model, such as the BBS models used annually by the CWS and USGS. Estimating annual indices as fixed-effects within each stratum greatly increases the overall number of parameters, which in turn affects precision. For BBS data, the number of parameters increases from 3 (mean and variance for trend, and variance of the year effects) to approximately 90 (mean and variance for each of the ~45 years in the time-series). As a compromise, we modelled the annual indices using a hierarchical structure in space (across strata, within each year), rather than in time (across years, within each stratum). We used a spatially explicit, conditional autoregressive error structure (spatial CAR) to model the cross-strata correlations in annual indices. The spatially explicit structure of the model retained much of the geographic variation in annual indices and trajectories while allowing the model to borrow information from neighbouring strata, thereby improving estimates for strata and years with relatively sparse data. We chose an explicitly spatial model because the spatial units are relatively small, in comparison to each species continental range (on average, 70 strata included for each species, and >150 strata overall), and it is reasonable to assume that regional breeding populations do not operate in isolation, i.e., that there are broad-scale processes influencing North American AI populations [16], [18], [20]. The spatial smoothing of the CAR model should not impose any spatial synchrony in the group-level change points that were our primary interest for two reasons: 1) the spatial smoothing in the CAR model is fit independently among species; and 2) the change point model considers the precision of each species’ annual indices, which will be relatively low for species*region combinations where the spatial smoothing is particularly influential. Our spatial CAR model is very similar to another recently described, spatially explicit model for the BBS [10], though there are some important differences, which we discuss in S2 Text.

The spatial CAR model accounts for observer effects and other nuisance parameters that are often modeled in estimating trends and trajectories derived from BBS data (e.g., [5], [6]). In the model, counts from a given BBS route, by a given observer (i.e., observer-route combinations, *j*), in year (*t*) and stratum (*i*) are assumed to have come from a Poisson distribution with mean (*λ*_*i*,*j*,*t*_).

$$\text{log}\;\left(\lambda_{i,j,t}\right)=s_{i}+\mu_{t}+\gamma_{i,t}+\omega_{i,j}+\zeta I\left(j,t\right)+\varepsilon_{i,j,t}$$ (1)

On the log-scale, the *λ* s were modeled by first-year observer effects (*ζ* **I**(*j*, *t*)), as well as mean-zero, normally distributed random effects for overdispersion (*ε*_*i*,*j*,*t*_) and observer-route effects (*ω*_*i*,*j*_). We included *s*_*i*_, the log of the observed average count across all routes and years in each stratum, as an offset in the model. We did this so that the spatial, year effects would model annual variation separately from variation in abundance among strata. We did not estimate *s*_*i*_ because the combination of *s*_*i*_, *μ*_*t*_, and *γ*_*i*,*t*_ is not separately estimable for most species and strata.

The main parameters that model population trajectories are year effects (*γ*_*i*,*t*_), which are drawn from separate distributions for each year, and which assume a first-order, intrinsic conditional autoregressive structure among neighbouring strata. We used the “car.normal” function in WinBUGS [22], [23] to define the autoregressive structure, correlating the year effects at time (*t*), in stratum (*i*) (*γ*_*i*,*t*_), with the average year effect at time (*t*) in all (*N*) neighbouring strata.

$$\gamma_{i,t}\propto Normal\;\left(\frac{1}{N}\left[\sum_{n=1}^{N}\gamma_{n,t}\right],\sigma_{t}\right)$$ (2)

Strata that share a border were considered to be neighbours. In the rare case where a stratum shared no borders with any other strata in the analysis (because of intervening strata with insufficient data for analysis), the isolated stratum was considered neighbouring with any other strata in the same BCR, or failing that, with the closest stratum (based on Euclidean distances between strata centroids). This was done to avoid removing these strata from the analyses, and under the assumption that population fluctuations in these putative edge-of-range strata are also closely linked to population fluctuations in nearby regions, closer to the core of the species’ range.

The average abundance for each year (*μ*_*t*_) was given a “flat” prior [23]. Priors for all other parameters were standard, non-informative conjugate priors, similar to those used [5] and further described in [6]. Briefly, all variances were assigned diffuse inverse gamma prior distributions (scale and shape parameters set to 0.001) and the parameter *ζ* was given a diffuse normal distribution (mean 0, variance 10^6^)

The annual indices (*η*_*i*,*t*_) of relative abundance for stratum (*i*) and year (*t*) are, following [5], exponentiated sums of the year, stratum, and trend effects, scaled by *π*_*i*_, the proportion of routes in the stratum on which the species was observed.

$$\eta_{i,t}=\pi_{i}*e^{S_{i}+\beta_{i}*t+\gamma_{i,t}+0.5*\sigma_{\omega_{i}}^{2}+0.5*\sigma_{\varepsilon }^{2}}$$ (3)

The variance components ($0.5*\sigma_{\omega_{i}}^{2}+0.5*\sigma_{\varepsilon }^{2}$) were added to correct for retransformation bias [6],[5].

### Identifying change points in trends

Using the annual indices from the spatial CAR model as data, we used hierarchical Bayesian, change point modeling techniques [3], [4], to estimate the number and timing of changes in the group-level trends of North American AI. The hierarchical structure of the change point model allowed us to estimate process variation (i.e., true population fluctuations) while accounting for observation error (i.e., the uncertainty around estimates of the annual indices), and to simultaneously model both group-level and species-specific change points and trajectories. The annual indices were log-transformed and then centered on their value in the mid-year of the time series for each stratum, so that all species’ indices were expressed on a common scale. The change point model estimated the number and location (i.e., the year) of change points in log-linear trends in the abundance of species and the group.

To account for error in their estimation, log-transformed and centered annual indices (*z*_*i*.*t*.*s*_) for species (*s*), year (*t*), and stratum (*i*) were assumed to be realisations of the true relative annual abundance (H_*i*,*t*,*s*_), which was modeled as the mean of a lognormal distribution. The variance of the lognormal distribution ($\sigma_{\eta_{i,t,s}}^{2}$) was not estimated in the model. Instead, estimates of the variance of the annual indices from the index model were used as data.

$$z_{i.t.s}=Normal\;\left({\text{H}}_{i,t,s},\sigma_{\eta_{i,t,s}}^{2}\right)$$

In the models that included a group trajectory (i.e., group-level change points, see Table 2), the true relative annual abundance of each species, on the log-scale (H_*i*,*t*,*s*_), was modeled as an additive function of two smoothed population trajectories: a group trajectory (e.g., *f*_*AllAI*_ (*t*) in the M_AllAI_ model) and a species trajectory (*f*_*s*_ (*t*), Table 2).

In addition to a model that assumed a common group-level change point across all aerial insectivores (M_AllAI_, Table 2), we fit three other models that included one or more group change points: M_FC_ with a group change point for flycatchers only, M_SSN_ with a group change point for swallows, swifts, and nightjars only, and M_FC,SSN_ with separate change points for both groups (Table 2). We also fit a model (M_S_) that did not include a group change point (Table 2). Within each stratum, we used the DIC [24] to compare the relative support for each of the 5 different models.

The smoothed trajectories (all *f* (*t*)) were estimated using a Bayesian piecewise linear regression spline model, with an unknown number of change points. Estimating an unknown number of change points requires varying the dimension of the model (i.e., the number of parameters to be estimated) among iterations of the Markov chain Monte Carlo (MCMC) sampling. We therefore implemented this model using reversible jump MCMC [25] in WinBUGS. At each step in the MCMC chain, a piecewise linear regression was fit, which was composed of a series of linear slopes joined at the estimated change points. The number and location of change points, as well as the slopes of the intervening linear trends, varied among steps in the MCMC chain. The full posterior distributions of slopes and change points combined to produce the regression spline that was the smoothed population trajectory. The posterior distributions provided estimates of the probabilities of change points occurring in particular years, while accounting for uncertainties in parameter estimates and model structure (i.e., the number of change points). The probability of a change point in any given year was the summed posterior probabilities of all models that include a change point in that year. Process error (i.e., annual fluctuations in population) around the smoothed spline trajectories was estimated as $\sigma_{p}^{2}$.

The species-specific and group-level smoothed population trajectories were fit separately within each stratum, using the following models (note: the *i* subscripts, indicating trajectories are stratum-specific, were suppressed to simplify the following equations):

$$f_{s}\left(t\right)=\beta_{s1}t+\sum_{j=1}^{k_{\beta_{s}}}\beta_{s}{}_{\left[j+1\right]}\text{I}\left(t\geq \theta_{s_{j}}\right)\left(t-\theta_{s_{j}}\right)$$

$$f_{AllAI}\left(t\right)=\beta_{AllAI_{1}}t+\sum_{j=1}^{k_{\beta_{AllAI}}}\beta_{AllAI}{}_{\left[j+1\right]}\text{I}\left(t\geq \theta_{AllAI_{j}}\right)\left(t-\theta_{AllAI_{j}}\right)$$

For each of the species trajectories, as well as for the group trajectory, the piecewise regression spline consisted of a collection of *k* + 1 slope parameters (*β*), joined at *k* change points (*θ*). The I(*t* ≥ *θ*_.*j*_) terms in the above equations are indicator variables that equal 1, if the condition inside the brackets is met and 0 otherwise.

The number of change points in each model (*k*_*β*._) were given binomial prior distributions that allowed for a maximum of 4 change points for a given species and stratum. In models that included a group trajectory, these 4 change points were split evenly between the group trajectory (*k*_*β*._ = *Binomial* (2, 0.5)), and each species trajectory ($k_{\beta_{s}}=Binomial\;\left(2,0.5\right)$). In models that did not include a group trajectory for a given species (e.g., for any flycatcher species, M_s_ and M_ssn_), the binomial prior for the number of change points was *k*_*β*._ = *Binomial* (4, 0.5). We included 0 to account for the possibility that the population trend was constant throughout the entire time series, and we chose 4 as the maximum number of change points to highlight the strongest changes in trends. The prior distributions were uninformative on the timing of individual change points, with the prior probability of a group trajectory change point occurring in any given year equal to 0.024 [*p*_0_ = (0.5 * 2)/41]. We considered strong evidence of a change point occurring in any given year to be a 3-fold (or greater) increase in the ratio of posterior odds [*p*_1_ / (1 − *p*_1_)] to prior odds [*p*_0_ / (1 − *p*_0_)].

We considered a posterior:prior odds ratio > 3, in any given year, as strong evidence in support of a trend change point. For regions and groups with these well-supported change points, we defined the timing of the change point, as the year with the highest odds ratios and therefore the highest posterior probability of including the change point. We indexed uncertainty in the specific timing of well-supported change points by counting the number of adjacent years that also had strong evidence for a change. As a measure of the overall support in the data for a given change point, we summed the year-specific posterior probabilities across all years in a sequence of years in which a particular group-level change point was strongly supported. Higher values of this metric imply that the existence of a change point is well supported both in situations where a single year has a relatively high posterior probability of including a change point and in situations where a sequence of years all have moderately high posterior probabilities of including the change point.

Simultaneously modeling group change points (i.e., *f*_*i*,*AllAI*_ (*t*), *f*_*i*,*FC*_ (*t*), or *f*_*i*,*SSN*_ (*t*)) and species change points (*f*_*i*,*s*_ (*t*)) allowed us to identify change points that were common across species, while accounting for change points that were only evident for a single species. To summarize and compare the change points across North America, we classified change points as either negative or positive. Negative change points were years during which the slope of the line decreased (e.g., a downturn from an increasing trend to stable, stable trend to decreasing, or decreasing trend to a more steeply decreasing trend). Positive change points were years during which the slope of the line increased, i.e., an upturn.

In theory, the two modeling processes (i.e., the spatial CAR model and the change point model) could be combined into a single model. In practice, computational limits (processing time and available memory) forced us to separate the two modeling processes. A benefit to this approach is that the index model produces annual indices that are not smoothed over time by any assumption of an underlying long-term trend. Therefore, these annual indices lend themselves well to additional modeling exercises focusing on spatial and temporal covariates of population fluctuations, as well as for visualizing regional patterns of population change. We have included all annual indices from the index model (S2 Table), as well as the BUGS language description of the spatial CAR model (S1 Text) and the regression spine model (S3 Text) to encourage further analyses.

## Supporting Information

S1 Fig Summary of the posterior probabilities of group-level change points. Box and whisker plots comparing the summed posterior probabilities of a negative or positive group-level change point, for strata in which the relevant change-point was well supported in at least one year of a particular decade. Colours match those used to indicate decades in Figs 3 and 5 of the original article. Numbers along the x-axis indicate the number of strata included in the associated box (i.e., the number of strata with a well-supported group-level change point in a particular decade). FC and SSN are two subgroups of avian aerial insectivores, and stand for Flycatchers and Swallows, Swifts, and Nightjars respectively.
(DOCX)

S2 Fig Overlapped range maps for 14 flycatchers. Overlapped breeding (A) and wintering (B) range maps for 14 flycatcher species included in our change point modeling. There is a relatively clear change in species composition from west to east during the breeding season, with strong separation of species primarily distributed in the east and those primarily distributed in the west. Wintering ranges of western breeding and eastern breeding species are much less separated. The change in species composition may partially explain the west/east split in timing of positive change points for flycatchers (Fig 5B). Species were classified based on the majority of their breeding range: Eastern species–Alder Flycatcher, Great Crested Flycatcher, Acadian Flycatcher, Yellow-bellied Flycatcher, Eastern Phoebe, Eastern Wood-Pewee and Eastern Kingbird; Western species–Western Kingbird, Says’ Phoebe, Pacific-slope Flycatcher, Western Wood-Pewee, and Olive-sided Flycatcher. Two species (Least Flycatcher and Willow Flycatcher) were classified as continental because their breeding ranges were approximately equally distributed across the continent.
(DOCX)

S1 Table Stratum-level supplementary material. Including model DIC values and timing of group-level change points.
(XLSX)

S2 Table Species-level supplementary material. Including timing of species-specific change points and the spatial CAR annual indices of abundance estimated for all species and strata.
(XLSX)

S1 Text WinBUGS spatial CAR model. Annual index model for the North American Breeding Bird Survey, with spatial conditional autoregressive (CAR) structure on stratum-level annual indices.
(TXT)

S2 Text Contrasting the spatial CAR model with Bled model. (DOCX)

S3 Text WinBUGS regression spline model MsubFCSSN. Bayesian hierarchical regression spline model used to identify both group-level and species-specific change points in population trends.
(TXT)

## Abbreviations

AI: aerial insectivores

BBS: North American Breeding Bird Survey

BCR: Bird Conservation Region

CAR: conditional autoregressive

CWS: Canadian Wildlife Service

DIC: Deviance Information Criterion

FC: flycatcher

M_AllAi_: all aerial insectivores model

MCMC: Markov Chain Monte Carlo

M_FC,SSN_: distinct flycatcher group and swallow, swift, and nightjar group model

M_FC_: flycatcher group model

M_SSN_: swallow, swift and nightjar group model

SSN: swallows, swifts and nightjars

USGS: United States Geological Survey
