# Supplementary material for: Change Points in the Population Trends of Aerial-Insectivorous Birds in North America: Synchronized in Time across Species and Regions
Source: PLoS One. 2015 Jul 6;10(7):e0130768. doi: 10.1371/journal.pone.0130768 (PMC4493114; doi:10.1371/journal.pone.0130768)
Supplement: S1 Fig — Box and whisker plots comparing the summed posterior probabilities of a negative or positive group-level change point, for strata in which the relevant change-point was well supported in at least one year of a particular decade. Colours match those used to indicate decades in Figs 3 and 5 of the original article. Numbers along the x-axis indicate the number of strata included in the associated box (i.e., the number of strata with a well-supported group-level change point in a particular decade). FC and SSN are two subgroups of avian aerial insectivores, and stand for Flycatchers and Swallows, Swifts, and Nightjars respectively. (DOCX) [file pone.0130768.s001.docx]

**
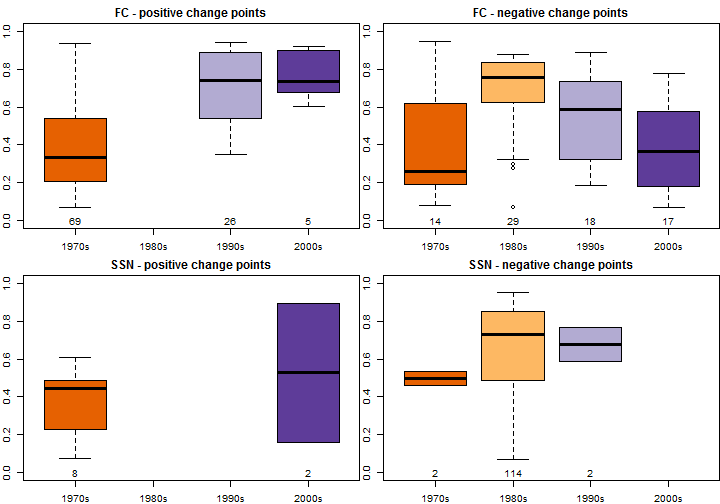
**

**S3 Fig. Summary of the summed posterior probabilities of well-supported group-level change points by decade.** Box and whisker plots comparing the summed posterior probabilities of a negative or positive group-level change point, for strata in which the relevant change-point was well supported in at least one year of a particular decade. Colours match those used to indicate decades in Figs. 3 and 5 of the original article. Numbers along the x-axis indicate the number of strata included in the associated box (i.e., the number of strata with a well-supported group-level change point in a particular decade). FC and SSN are two subgroups of avian aerial insectivores, and stand for Flycatchers and Swallows, Swifts, and Nightjars respectively.
