# Supplementary material for: Change Points in the Population Trends of Aerial-Insectivorous Birds in North America: Synchronized in Time across Species and Regions
Source: PLoS One. 2015 Jul 6;10(7):e0130768. doi: 10.1371/journal.pone.0130768 (PMC4493114; doi:10.1371/journal.pone.0130768)
Supplement: S2 Fig — Overlapped breeding (A) and wintering (B) range maps for 14 flycatcher species included in our change point modeling. There is a relatively clear change in species composition from west to east during the breeding season, with strong separation of species primarily distributed in the east and those primarily distributed in the west. Wintering ranges of western breeding and eastern breeding species are much less separated. The change in species composition may partially explain the west/east split in timing of positive change points for flycatchers (Fig 5B). Species were classified based on the majority of their breeding range: Eastern species–Alder Flycatcher, Great Crested Flycatcher, Acadian Flycatcher, Yellow-bellied Flycatcher, Eastern Phoebe, Eastern Wood-Pewee and Eastern Kingbird; Western species–Western Kingbird, Says’ Phoebe, Pacific-slope Flycatcher, Western Wood-Pewee, and Olive-sided Flycatcher. Two species (Least Flycatcher and Willow Flycatcher) were classified as continental because their breeding ranges were approximately equally distributed across the continent. (DOCX) [file pone.0130768.s002.docx]

**
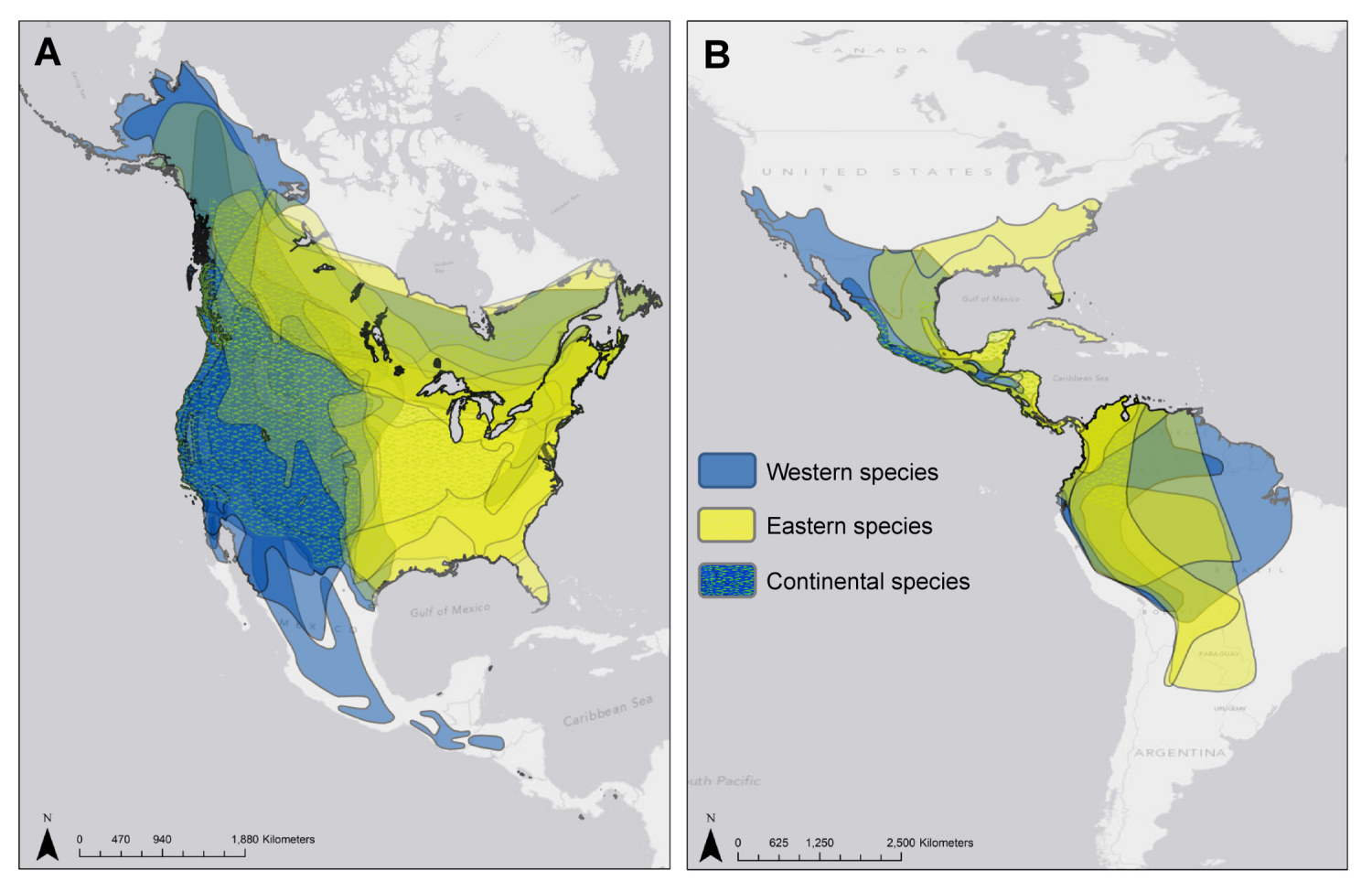
**

**S4 Fig. Overlapped range maps for 14 flycatchers.** Overlapped breeding (A) and wintering (B) range maps for 14 flycatcher species included in our change point modeling. There is a relatively clear change in species composition from west to east during the breeding season, with strong separation of species primarily distributed in the east and those primarily distributed in the west. Wintering ranges of western breeding and eastern breeding species are much less separated. The change in species composition may partially explain the west/east split in timing of positive change points for flycatchers (Fig. 5B). Species were classified based on the majority of their breeding range: Eastern species – Alder Flycatcher, Great Crested Flycatcher, Acadian Flycatcher, Yellow-bellied Flycatcher, Eastern Phoebe, Eastern Wood-Pewee and Eastern Kingbird; Western species – Western Kingbird, Says’ Phoebe, Pacific-slope Flycatcher, Western Wood-Pewee, and Olive-sided Flycatcher. Two species (Least Flycatcher and Willow Flycatcher) were classified as continental because their breeding ranges were approximately equally distributed across the continent.
