# Supplementary material for: Change Points in the Population Trends of Aerial-Insectivorous Birds in North America: Synchronized in Time across Species and Regions
Source: PLoS One. 2015 Jul 6;10(7):e0130768. doi: 10.1371/journal.pone.0130768 (PMC4493114; doi:10.1371/journal.pone.0130768)
Supplement: S2 Text — (DOCX) [file pone.0130768.s006.docx]

Contrasting the spatial CAR model used in Smith et al. with the one used in Bled et al. 2013

The spatial CAR annual index model that we have described here is very similar to the model described in [1], hereafter the Bled model. In fact, the major difference is that the models are applied at different spatial grains (degree block grid versus the analytical strata used here). Although described in the paper as a “trend model”, by our definition here, the Bled model is an annual index model (i.e., it does not fit a long-term, smooth or linear trend). Another difference is that our model, following [2], estimates the variance in observer-route effects separately for each stratum, by assuming they are drawn from a distribution of variances with a common mean and variance across all strata. In the Bled model, the observer-route effects are drawn from a common distribution across all strata, with a single, survey-wide estimate of their variance. This difference in the way observer-route effects are structured can have important effects on the estimated trends from alternative trend models [2]. The second difference is that the spatial CAR model has a stratum-specific intercept that is not estimated but instead entered as an offset. This intercept has the effect of accounting for much of the variation among strata in overall abundance of each species, which therefore reduces the component of the variation in abundance that remains to be modeled by the spatial autoregressive effects. That is, for any given year, the spatial effects described here only model departures from a stratum’s mean abundance; they do not model variations among strata in overall abundance. In the Bled model, the spatial effects account for the variation in overall abundance among strata. This difference would also interact with the differences in the structure of the observer-route effects, and the different stratifications, in ways that are difficult to predict.

References

1. Bled F, Sauer J, Pardieck K, Doherty P, Royle JA (2013) Modeling trends from North American Breeding Bird Survey data: A spatially explicit approach. PLoS ONE 8: e81867. Available: <http://www.plosone.org/article/info%3Adoi%2F10.1371%2Fjournal.pone.0081867> (Accessed 7 November 2014)
2. Smith AC, Hudson M-AR, Downes CM, Francis CM (2014) Estimating breeding bird survey trends and annual indices for Canada: how do the new hierarchical Bayesian estimates differ from previous estimates. Canadian Field-Naturalist 128: 119-134. Available: <http://www.canadianfieldnaturalist.ca/index.php/cfn/article/view/1565> (Accessed 7 November 2014)
